# Rifampicin Resistance Associated with *rpoB* Mutations in Neisseria gonorrhoeae Clinical Strains Isolated in Austria, 2016 to 2020

**DOI:** 10.1128/spectrum.02757-21

**Published:** 2022-06-09

**Authors:** Justine Schaeffer, Kathrin Lippert, Sonja Pleininger, Anna Stöger, Petra Hasenberger, Silke Stadlbauer, Florian Heger, Angelika Eigentler, Alexandra Geusau, Alexander Indra, Franz Allerberger, Werner Ruppitsch

**Affiliations:** a Institute for Medical Microbiology and Hygiene, Austrian Agency for Health and Food Safety, Vienna, Austria; b EUPHEM Fellowship, European Centre for Disease Prevention and Control (ECDC), Stockholm, Sweden; c MB-LAB - Clinical Microbiology Laboratory, Innsbruck, Austria; d Department of Dermatology, Medical University of Vienna, Vienna, Austria; e Paracelsus Medical University of Salzburg, Salzburg, Austria; f Department of Biotechnology, University of Natural Resources and Life Sciences, Vienna, Austria; University at Albany, State University of New York

**Keywords:** *Neisseria gonorrhoeae*, antibiotic resistance, rifampicin, whole-genome sequencing

## Abstract

Due to increasing rates of antimicrobial resistance (AMR) in Neisseria gonorrhoeae, alternative treatments should be considered. To assess rifampicin’s potential as a gonorrhea treatment, we used *rpoB* mutations to estimate rifampicin resistance in Austrian N. gonorrhoeae isolates. We found 30% of resistant isolates clustering in three main phylogenomic branches. Rifampicin resistance was associated with resistance to other antibiotics. Therefore, rifampicin cannot be recommended as an alternative gonorrhea treatment in Austria, even in combination therapy.

**IMPORTANCE** Gonorrhea, caused by Neisseria gonorrhoeae, is one of the most common bacterial sexually transmitted infections. It is treated with antibiotics, but an increasing number of N. gonorrhoeae strains are resistant to currently used treatments. In this study, we explored the potential of rifampicin, another antibiotic, as a treatment option for gonorrhea. However, around 30% of Austrian N. gonorrhoeae strains investigated were already resistant to rifampicin, which would limit its benefit as a gonorrhea treatment.

## INTRODUCTION

Gonorrhea is the second most common bacterial sexually transmissible infection in the EU and worldwide (1). Gonorrhea is treated with antibiotics; however, Neisseria gonorrhoeae has developed or acquired antimicrobial resistance (AMR) to all antimicrobials previously recommended for empirical treatment of gonorrhea (2). Therefore, alternative therapies should be considered. Here, we studied the potential efficacy of rifampicin. Rifampicin is one of the most potent broad-spectrum antibiotics. Although clinical trials more than 30 years ago showed its efficacy in treating gonorrhea, rifampicin has never been established in therapy guidelines (3, 4). However, the potential of this treatment is conditioned by the preexistence of rifampicin resistance in the N. gonorrhoeae population. In Mycobacterium tuberculosis, 95% of rifampicin resistance is caused by single nucleotide variants (SNVs) in the *rpoB* gene, coding for the β subunit of the RNA polymerase (5). Similar impacts of *rpoB* mutations on rifampicin resistance have been highlighted for Mycobacterium leprae, Mycobacterium kansasii, and other bacterial species such as Escherichia coli, Bacillus subtilis, Staphylococcus aureus, and Neisseria meningitidis (6–8). Therefore, we decided to use these SNVs as a proxy to investigate the frequency of rifampicin resistance in Austrian N. gonorrhoeae clinical strains. In a context where multidrug resistance of N. gonorrhoeae poses a major threat of treatment failure, such data would help to evaluate the benefit of considering rifampicin as an alternative gonorrhea treatment.

## RESULTS

**FIG 1:**
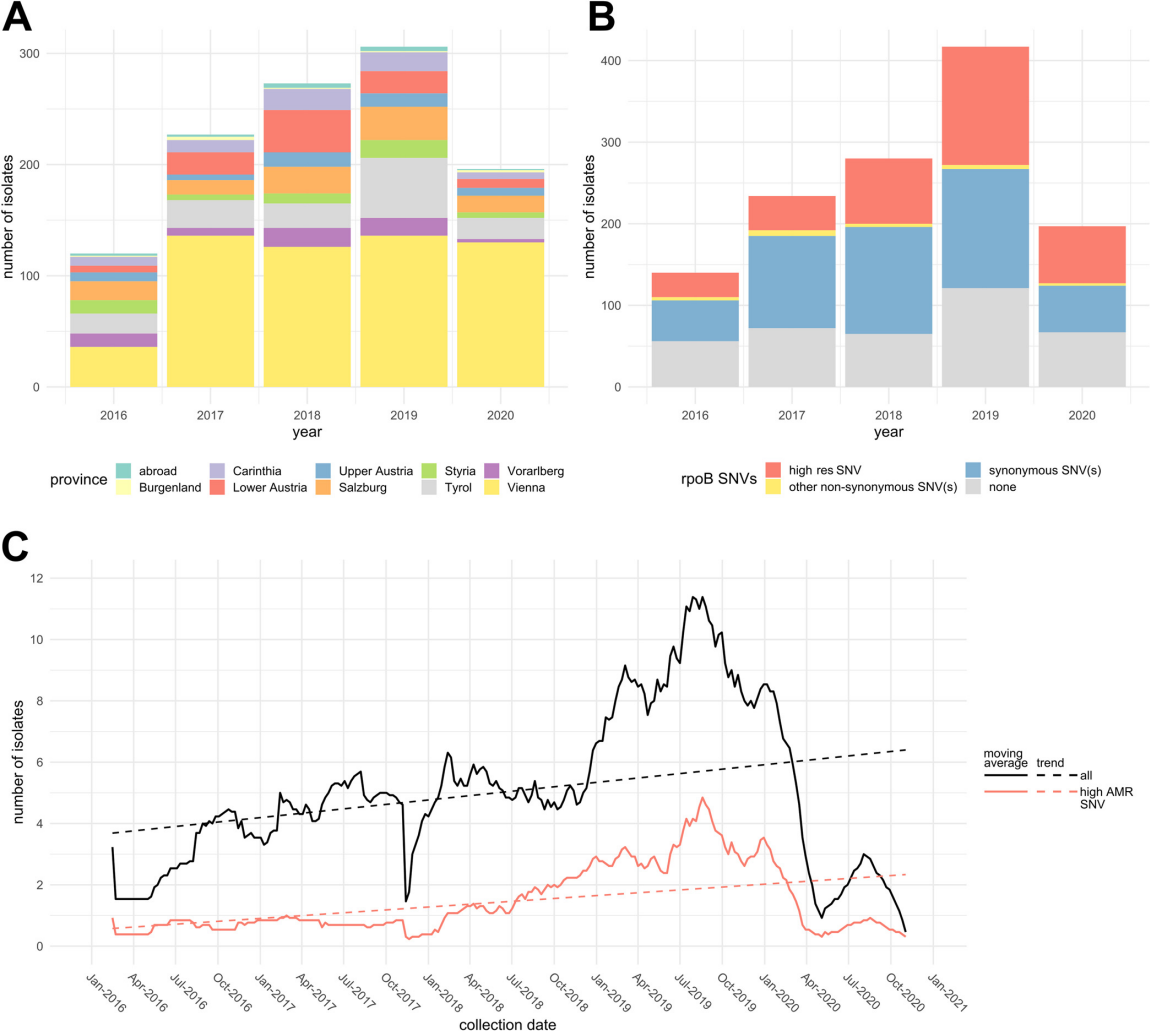
*rpoB* single nucleotide variants (SNV) found in the Austrian N. gonorrhoeae isolates. (A) Number of isolates according to the year of isolation and the province of origin (*n* = 1,268). (B) Number of isolates carrying *rpoB* SNVs associated with high levels of rifampicin resistance (high res SNV), other nonsynonymous SNVs, and synonymous SNVs, according to the year of isolation (*n* = 1,568). (C) Evolution of the number of resistant isolates over time. Solid lines indicate the 13-week moving average of the number of isolates classified as resistant (*n* = 1,268). Trends over time (obtained by linear regression) are represented by the dashed lines.

N. gonorrhoeae isolates from symptomatic and asymptomatic patients were either provided by 16 Austrian laboratories or cultured at the National Reference Centre (NRC) for Gonococci from clinical specimens (see Table S1 in the supplemental material). Phenotypic AMR was assessed with Etest (bioMérieux, Marcy l’Etoile, France, and Liofilchem, Roseto degli Abruzzi, Italy) according to EUCAST guidelines (9). Approximately 50% of the samples were collected in the province of Vienna, but the collection included isolates from all Austrian provinces (Fig. 1A).

Since 2016, whole-genome sequencing (WGS) has been performed on all isolates collected at the NRC. DNA extraction, library preparation, and Illumina sequencing have been described elsewhere (10). A total of 1,568 N. gonorrhoeae isolates collected between 2016 and 2020, for which sequencing data were available, were included in this study. Data on phenotypic AMR testing were available for 1,268 of them.

A reference *rpoB* sequence was obtained from the complete genome of N. gonorrhoeae strain 35/02 (accession no. NZ_CP012028.1). Isolates’ *rpoB* sequences were extracted from WGS data using SeqSphere+ (Ridom, Muenster, Germany), with a minimum of 99% alignment and 90% identity to the reference sequence. Extracted sequences were searched for SNVs with the SeqSphere+ tool, and 223 SNVs were found, including 33 nonsynonymous SNVs (Table 1). Thirty of 33 SNVs were found in only one or two isolates. Thirty-two isolates, one for each SNV combination that included at least one nonsynonymous SNV, were unfrozen and cultured. Rifampicin Etests (bioMérieux) were performed for the 26/32 viable isolates, and results are presented in Table 1. No results were obtained for 6 SNVs, and 20 SNVs were found only in isolates susceptible to rifampicin. Two SNVs were associated with a high level of rifampicin resistance (minimum inhibitory concentration [MIC] of >32 mg/L): C1657A/His553Asn and T1679C/Leu560Ser. These two SNVs correspond to His526Asn and Leu533Ser in E. coli and were described to confer rifampicin resistance in several bacterial species (11, 12). To assess the impact of genetic backgrounds on rifampicin resistance associated with C1657A, 32 isolates corresponding to 31 different sequence types were tested for rifampicin resistance. All of them had a MIC of >32 mg/L, confirming the association between C1657A and high levels of rifampicin resistance in N. gonorrhoeae. C1657A and T1679C were found in 473 isolates, which corresponded to 30% of the isolates (Fig. 1B). This proportion was stable over time (Fig. 1C). Five other nonsynonymous SNVs were associated with rifampicin MICs between 0.25 mg/L and 0.5 mg/L. However, given the absence of standardized thresholds for rifampicin resistance in N. gonorrhoeae, the possible involvements of other resistance genes, and the unknown clinical relevance, these SNVs were not analyzed any further in this study.

**TABLE 1:** Single nucleotide variants identified in the *rpoB* sequences of Austrian N. gonorrhoeae isolates (*n* = 1,568)^*a*^

Nucleotide							Amino acid							MIC (μg/mL)	No. of isolates tested
Position	Reference			Variant			Position	Reference			Variant				
	Base	No.	%	Base	No.	%		aa	No.	%	aa	No.	%		
5	A	1,567	99.9	G	1	0.1	2	N	1,567	99.9	S	1	0.1	0.19	1^*c*^
226	T	1,566	99.9	C	2	0.1	76	Y	1,566	99.9	H	2	0.1	0.19	2^*b*^^,^^*c*^
239	G	1,558	99.4	A	10	0.6	80	G	1,558	99.4	D	10	0.6	NA	NA
*404*	*C*	*1,567*	*99.9*	*T*	*1*	*0.1*	*135*	*T*	*1,567*	*99.9*	*I*	*1*	*0.1*	*0.38*	*1*
505	C	1,566	99.9	T	2	0.1	169	H	1,566	99.9	Y	2	0.1	0.19	1^*d*^
730	G	1,567	99.9	A	1	0.1	244	D	1,567	99.9	N	1	0.1	NA	NA
743	G	1,567	99.9	A	1	0.1	248	G	1,567	99.9	D	1	0.1	0.125	1
*824*	*C*	*1,566*	*99.9*	*T*	*2*	*0.1*	*275*	*T*	*1,566*	*99.9*	*I*	*2*	*0.1*	*0.38*	*1*
838	C	1,566	99.9	T	2	0.1	280	R	1,566	99.9	C	2	0.1	NA	NA
881	A	1,566	99.9	C	2	0.1	294	Q	1,566	99.9	P	2	0.1	0.19	2^*b*^^,^^*c*^
983	C	1,567	99.9	T	1	0.1	328	A	1,567	99.9	V	1	0.1	0.094	1
1094	A	1,566	99.9	C	2	0.1	365	Q	1,566	99.9	P	2	0.1	0.19	1^*d*^
1323	C	1,566	99.9	A	2	0.1	441	D	1,566	99.9	E	2	0.1	0.25	1
*1346*	*T*	*1,567*	*99.9*	*C*	*1*	*0.1*	*449*	*V*	*1,567*	*99.9*	*A*	*1*	*0.1*	*0.38*	*1*
**1657**	**C**	**1,096**	**69.9**	**A/T**	**472**	**30.1**	**553**	**H**	**1,096**	**69.9**	**N/Y**	**472**	**30.1**	**>32**	**32**
**1679**	**T**	**1,567**	**99.9**	**C**	**1**	**0.1**	**560**	**L**	**1,567**	**99.9**	**S**	**1**	**0.1**	**>32**	**1**
1769	C	1,567	99.9	T	1	0.1	590	T	1,567	99.9	M	1	0.1	0.064	1
2018	C	1,567	99.9	T	1	0.1	673	T	1,567	99.9	M	1	0.1	0.19	1^*b*^
2048	C	1,567	99.9	T	1	0.1	683	A	1,567	99.9	V	1	0.1	0.125	1
2075	C	1,567	99.9	T	1	0.1	692	A	1,567	99.9	V	1	0.1	NA	NA
2146	G	1,567	99.9	A	1	0.1	716	A	1,567	99.9	T	1	0.1	0.125	1
2309	C	1,567	99.9	T	1	0.1	770	A	1,567	99.9	V	1	0.1	0.25	1
*2405*	*A*	*1,566*	*99.9*	*G*	*2*	*0.1*	*802*	*D*	*1,566*	*99.9*	*G*	*2*	*0.1*	*0.5*	*1*
3169	T	1,566	99.9	G	2	0.1	1057	L	1,566	99.9	V	2	0.1	0.19	2^*b*^^,^^*c*^
*3523*	*G*	*1,567*	*99.9*	*A*	*1*	*0.1*	*1175*	*E*	*1,567*	*99.9*	*K*	*1*	*0.1*	*0.38*	*1*
3525	G	1,567	99.9	C	1	0.1	1175	E	1,567	99.9	D	1	0.1	NA	NA
3527	G	1,566	99.9	A	2	0.1	1176	R	1,566	99.9	Q	2	0.1	0.19	2^*b*^^,^^*c*^
3563	A	1,567	99.9	G	1	0.1	1188	K	1,567	99.9	R	1	0.1	0.19	1^*c*^
3601	A	1,567	99.9	G	1	0.1	1201	S	1,567	99.9	G	1	0.1	0.19	1^*b*^
3602	G	1,567	99.9	C	1	0.1	1201	S	1,567	99.9	T	1	0.1	0.19	1^*b*^
3732	A	1,566	99.9	C/G	2	0.1	1244	E	1,567	99.9	D	1	0.1	0.25	1
3800	C	1,566	99.9	T	2	0.1	1267	S	1,566	99.9	L	2	0.1	0.19	1
3937	G	1,565	99.8	A	3	0.2	1313	A	1,565	99.8	T	3	0.2	NA	NA

a For each variant, this table gives the number and frequency (%) of isolates carrying the reference and variant nucleotide/amino acid. Results of rifampicin Etests and the number of tested isolates are indicated in the last columns. SNVs associated with high or low levels of rifampicin resistance are indicated in boldface and italic, respectively. aa, amino acid; NA, not available.

b One isolate containing SNVs 226, 881, 2018, 3169, 3527, 3601, and 3602 with a MIC of 0.19 μg/mL.

c One isolate containing SNVs 5, 226, 881, 3169, 3527, and 3563 with a MIC of 0.19 μg/mL.

d One isolate containing SNVs 505 and 1094 with a MIC of 0.19 μg/mL.

**FIG 2:**
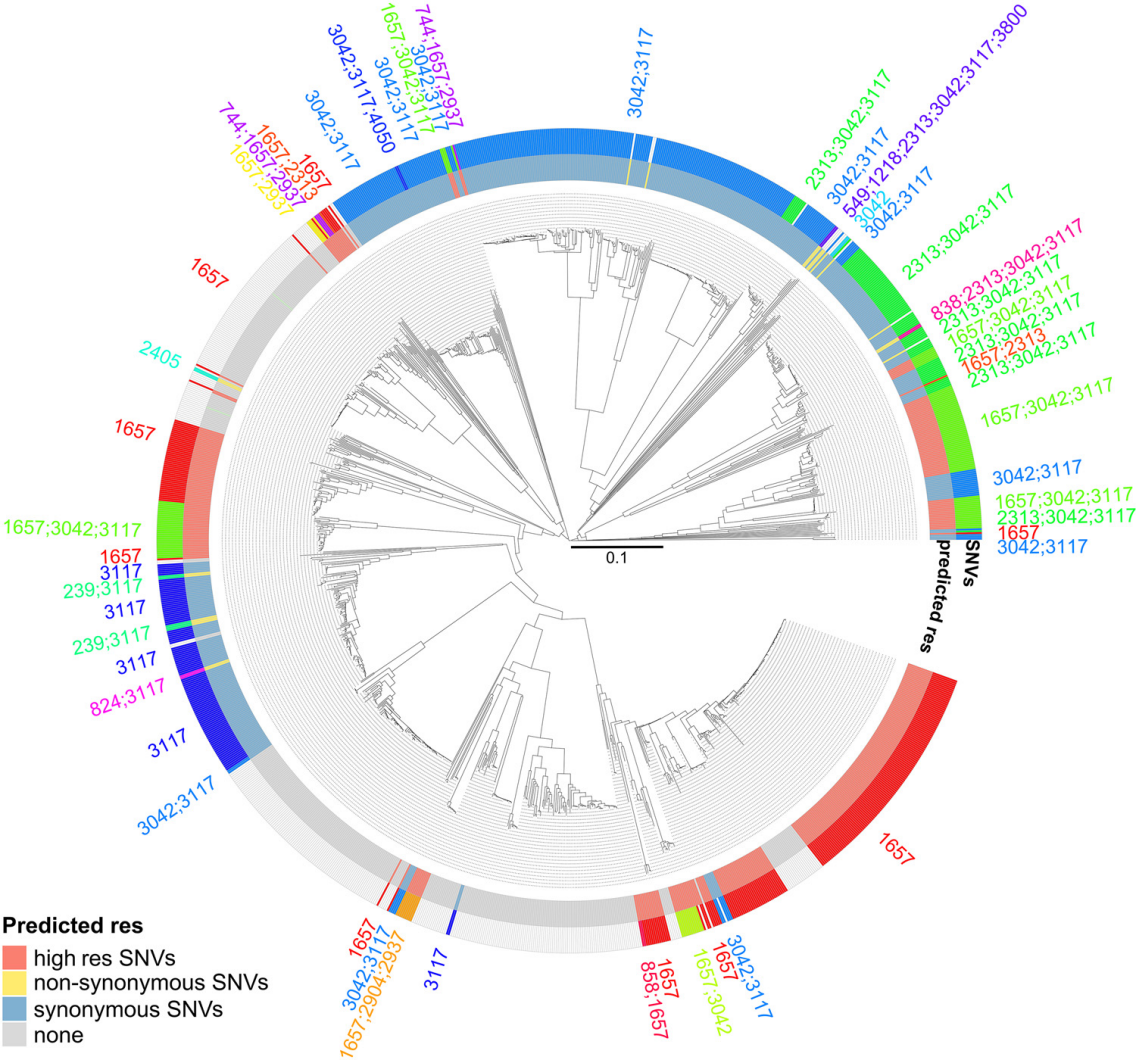
Correlation between population structure and *rpoB* single nucleotide variants (SNVs). The dendrogram was computed from the distance matrix of the core genome MLST (cgMLST) analysis (*n* = 1,568). Branch length indicates the proportion of different alleles in cgMLST between two isolates. The outer rims indicate the combination of *rpoB* SNVs and the predicted rifampicin AMR according to these SNVs. High res SNVs, at least one mutation conferring a high level of rifampicin resistance; nonsynonymous SNVs, at least one nonsynonymous mutation but no mutation conferring a high level of rifampicin resistance; synonymous SNVs, at least one synonymous SNV and no nonsynonymous SNVs; none, no SNVs compared with reference sequence (NZ_CP012028.1).

Phylogenomic relatedness between the isolates was assessed using a local core genome multilocus sequence typing (MLST) scheme previously described (13). The distribution of the isolates was compared with the combination of SNVs carried by their *rpoB* gene and their potential impact on rifampicin resistance (Fig. 2). Isolates with similar SNV combinations grouped in close branches. Particularly, isolates carrying C1657A or T1679C (indicated as “high-AMR SNVs”) were found in three main branches. This correlation between *rpoB* variants and population structure suggested a limited number of introductions of rifampicin resistance in Austrian N. gonorrhoeae strains and a mainly vertical evolution of the *rpoB* gene.

**FIG 3:**
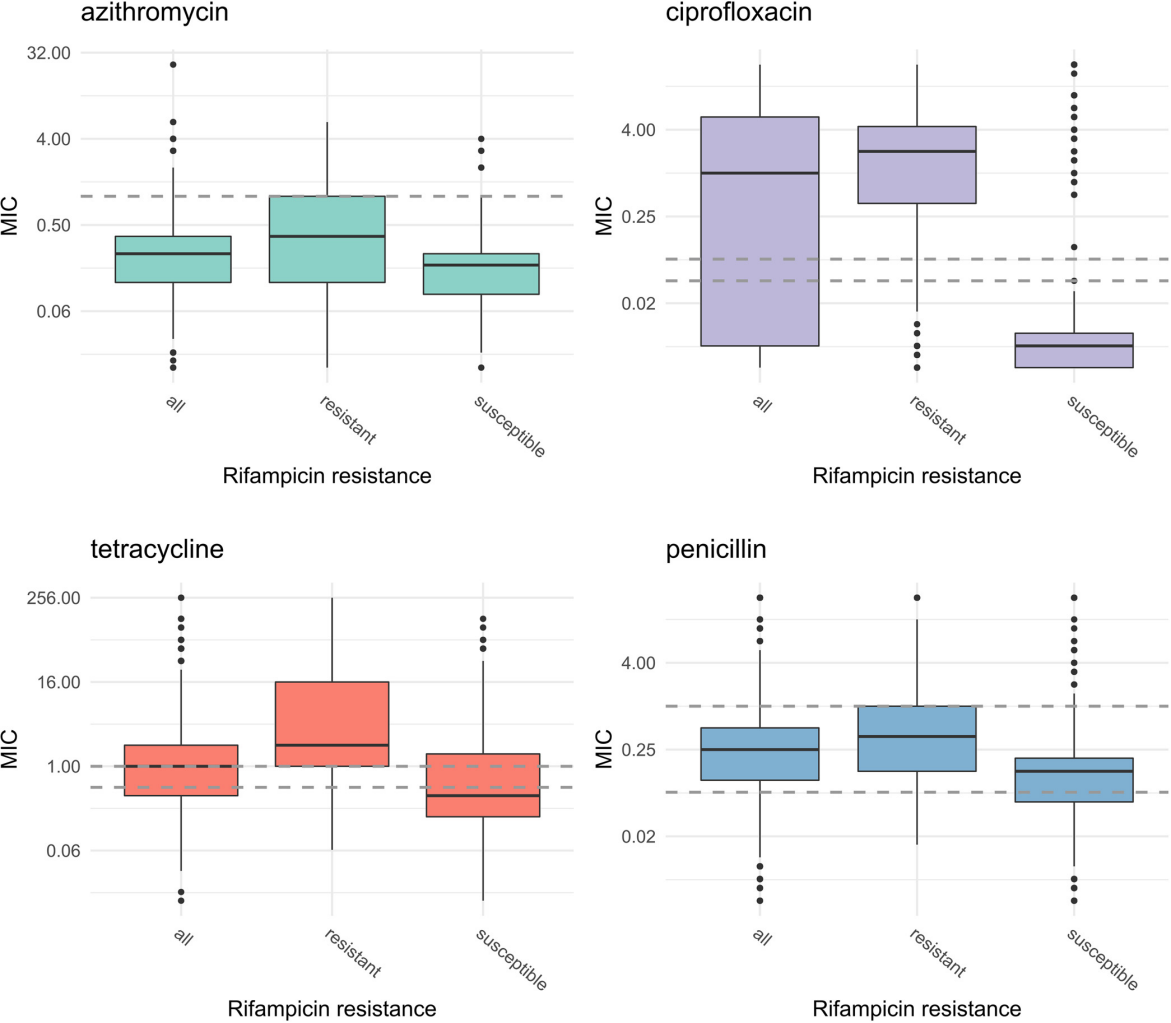
Correlation between MIC and *rpoB* single nucleotide variants (SNVs) associated with rifampicin resistance (*n* = 1,268). Boxplots represent the distribution of MICs obtained by Etest for all study isolates (“all”) and isolates carrying *rpoB* with C1657A or T1679C (“resistant”) and without these SNVs (“susceptible”). Dashed lines indicate the thresholds used to classify the isolates as susceptible/intermediate/resistant for ciprofloxacin, tetracycline, and penicillin and as susceptible/resistant for azithromycin.

The association between rifampicin resistance and resistance to other antimicrobials was tested using univariate analysis. Measures of association (odds ratio [OR]) and significance of the association (*P* value < 0.05) are shown in Table 2. Isolates carrying *rpoB* mutations associated with rifampicin resistance were more likely to be resistant to azithromycin (OR = 22 [95% confidence interval, 10–48.1]), ciprofloxacin (OR = 15 [10.7–20.9]), penicillin (OR = 4.28 [2.74–6.7]), and tetracycline (OR = 7.62 [5.55–10.5]) (Table 2 and Fig. 3).

**TABLE 2:** Association of AMR with rifampicin resistance in N. gonorrhoeae isolates (*n* = 1,268)^*a*^

Antimicrobial and category	Rifampicin resistant			Rifampicin susceptible			Univariate analysis	
	Total no.	No.	%	Total no.	No.	%	OR (95% CI)	*P* value
Azithromycin								
Resistant	361	84	23.3	514	7	1.4	22 (10–48.1)	<0.0001
Susceptible	361	277	76.7	514	507	98.6	0.046 (0.021–0.1)	<0.0001

Ciprofloxacin								
Resistant	364	292	80.2	516	110	21.3	15 (10.7–20.9)	<0.0001
Susceptible	364	72	19.8	516	406	78.7	0.067 (0.048–0.093)	<0.0001

Penicillin								
Resistant	364	76	20.9	517	30	5.8	4.28 (2.74–6.7)	<0.0001
Intermediate	364	246	67.6	517	298	57.6	1.53 (1.16–2.03)	0.0031
Susceptible	364	42	11.5	517	189	36.6	0.226 (0.157–0.327)	<0.0001

Tetracycline								
Resistant	338	250	74	475	129	27.2	7.62 (5.55–10.5)	<0.0001
Intermediate	338	49	14.5	475	42	8.8	1.75 (1.13–2.71)	0.01305
Susceptible	338	39	11.5	475	304	64	0.073 (0.05–0.108)	<0.0001

a For each antimicrobial, number of resistant isolates, total number of isolates, and resistance frequency are indicated for rifampicin-resistant (carrying *rpoB* with C1657A or T1679C) and susceptible isolates. Odds ratio (OR) and 95% confidence interval (CI) were calculated, and association was tested with Fisher’s exact test.

## DISCUSSION

To summarize, we exploited a large collection of genomic data to evaluate rifampicin resistance in the Austrian N. gonorrhoeae population, using *rpoB* SNVs as a proxy for rifampicin resistance. Mutations C1657A and T1679C, described to confer high levels of rifampicin resistance, were found in 30% of the isolates. This rate was high, even though rifampicin is rarely used in Austria and therefore the selective pressure to maintain rifampicin resistance is low (14, 15). Phylogenomic analysis of the isolates supported the hypothesis of mainly vertical evolution of *rpoB* and limited introductions of rifampicin-resistant isolates. Furthermore, rifampicin resistance was strongly associated with other AMR. According to these findings, rifampicin should not be considered as an alternative treatment for gonorrhea in Austria, neither alone nor in combination.

## MATERIALS AND METHODS

### Samples.

N. gonorrhoeae isolates were collected and stored at the National Reference Centre (NRC) for Gonococci (see Table S1 in the supplemental material). These isolates were tested for phenotypic resistance to azithromycin, cefixime, ceftriaxone, ciprofloxacin, tetracycline, and benzylpenicillin using Etest (bioMérieux, Marcy l’Etoile, France, and Liofilchem, Roseto degli Abruzzi, Italy) at the NRC or at MB-LAB. MIC thresholds used in this study followed the EUCAST guidelines (9). For azithromycin, isolates were defined as susceptible for a MIC of ≤1 μg/mL and resistant for a MIC of >1 μg/mL, according to the EUCAST epidemiological cutoff.

### WGS.

Genomic DNA isolation, WGS, assembly, and contig filtering were performed as described previously (10). High-molecular-weight (HMW) DNA was isolated from cultures using the MagAttract HMW DNA kit (Qiagen, Hilden, Germany), following the manufacturer’s protocol for Gram-negative bacteria. Ready-to-sequence libraries were obtained with the NexteraXT kit (Illumina, Inc., San Diego, CA, USA). Paired-end sequencing (2 by 300 bp) was performed on a MiSeq instrument (Illumina). Raw reads were *de novo* assembled into a draft genome using SPAdes (version 3.11.1) (16). Contigs were filtered for a minimum coverage of 5 and minimum length of 200 bp. Sequencing quality was checked with FastQC. Sequencing generated 106,428 to 2,927,502 reads and a coverage of 15- to 272-fold.

### *rpoB* allele library.

An allele library including only the *rpoB* sequence from the complete genome of N. gonorrhoeae strain 35/02 (accession no. NZ_CP012028.1) was built on SeqSphere+. This library was used to identify *rpoB* genes in WGS data. Target sequence needed to have >99% alignment with the reference sequence, >90% identity, and no frameshift. Target sequences corresponding to the *rpoB* gene were extracted and searched for SNV using SeqSphere+. Nonsynonymous SNVs were compared with mutations described to confer rifampicin AMR in E. coli, M. tuberculosis, and N. meningitidis in the literature (6, 11, 12) or the DRAG database (17).

### cgMLST.

A local N. gonorrhoeae core genome MLST (cgMLST) scheme was generated with the SeqSphere+ target definer tool (version 6.0.0; Ridom, Muenster, Germany) (18) using strain MS11 as a seed genome (NCBI accession number NC_022240.1) and 47 complete N. gonorrhoeae genomes as query sequences (accession numbers NC_002946.2, NC_011035.1, NZ_CP012026.1, NZ_CP012027.1, NZ_CP012028.1, NZ_CP016015.1, NZ_CP016016.1, NZ_CP016017.1, ABZF00000000.1, ABZG00000000.1, ABZH00000000.1, ACIG00000000.1, ADAA00000000.1, ABZJ00000000.2, ABZI00000000.1, ABZM00000000.1, ABZL00000000.1, ABZN00000000.1, ABZO00000000.1, ABZP00000000.1, ABZQ00000000.1, CQLK00000000.1, CQJM00000000.1, CQME00000000.1, CQJI00000000.1, CQIM00000000.1, CQHK00000000.1, CQLD00000000.1, CQNW00000000.1, CQKW00000000.1, CQJY00000000.1, CQIY00000000.1, CQJB00000000.1, CQKU00000000.1, CQOV00000000.1, CQIR00000000.1, CQJZ00000000.1, CQKM00000000.1, CQMI00000000.1, CQMT00000000.1, CQKB00000000.1, CQOT00000000.1, CQJD00000000.1, CHZN00000000.1, CFRU00000000.1, AKCG00000000.1, and AKCH00000000.1) and applying default software parameters. A 1,524-locus cgMLST scheme and a 463-locus accessory target scheme were obtained, as described in a previous publication (13). Using the distance matrix of the cgMLST analysis, a neighbor-joining tree was computed using SeqSphere+ (Sattath and Tversky’s method). The computed tree was exported in Newick Tree Format and loaded into R to compute dendrograms (packages ggplot [19], ggpubr [20], ape [21], and ggtree [22]).

### Data availability.

Raw reads were deposited in the Sequence Read Archive (SRA) under project no. PRJNA771206.
